# Intracarotid administration of human bone marrow mononuclear cells in rat photothrombotic ischemia

**DOI:** 10.1186/2040-7378-2-3

**Published:** 2010-02-02

**Authors:** Jens Minnerup, Florian H Seeger, Katharina Kuhnert, Kai Diederich, Matthias Schilling, Stefanie Dimmeler, Wolf-Rüdiger Schäbitz

**Affiliations:** 1 Department of Neurology, University of Münster, Albert-Schweitzer-Straße 33, 48149 Münster, Germany; 2 Division of Cardiology, Department of Medicine, Goethe University, Theodor-Stern-Kai 7 D-60590 Frankfurt (Main) Germany, Germany; 3 Institute for Cardiovascular Regeneration, Centre of Molecular Medicine, Goethe University, Theodor-Stern-Kai 7 D-60590 Frankfurt (Main) Germany, Germany; 4 Department of Neurology, University of Münster and Evangelisches Krankenhaus Bielefeld, Burgsteig 13, 33617 Bielefeld, Germany

## Abstract

**Background:**

Increasing evidence suggests that cell therapy improves functional recovery in experimental models of stroke and myocardial infarction. So far only small pilot trials tested the effects of cell therapy in stroke patients, whereas large clinical trials were conducted in patients with ischemic heart disease. To investigate the therapeutic benefit of cell therapy to improve the recovery after stroke, we determined the efficacy of bone marrow derived mononuclear cells, which were shown to improve the recovery in experimental and clinical acute myocardial infarction studies, in a rat stroke model.

**Methods:**

Adult male Wistar rats were randomly assigned to receive either five million human bone marrow mononuclear cells (hBMC) or placebo intraarterially 3 days after photothrombotic ischemia. For immunosuppression the animals received daily injections of cyclosporine throughout the experiment, commencing 24 hours before the cell transplantation. A battery of behavioral tests was performed before and up to 4 weeks after ischemia.

**Results:**

Body temperature and body weight revealed no difference between groups. Neurological deficits measured by the Rotarod test, the adhesive-removal test and the cylinder test were not improved by hBMC transplantation compared to placebo.

**Conclusions:**

This study demonstrates that hBMC do not improve functional recovery when transplanted intraaterially 3 days after the onset of focal cerebral ischemia. A possible reason for the failed neurological improvement after cell therapy might be the delayed treatment initiation compared to other experimental stroke studies that showed efficacy of bone marrow mononuclear cells.

## Background

Stroke is the second leading cause of death after myocardial infarction and the most frequent cause of adult disability [1,2]. For both cardiovascular and cerebrovascular disease cell therapy emerged as a promising therapeutic option [3-5]. In animal models of focal cerebral ischemia and myocardial infarction cells of various sources were shown to improve outcome [6-11]. In contrast to clinical studies on ischemic heart disease, in which cell therapies were thoroughly investigated, only small pilot trials of cell treatment in stroke patients were performed [12,13]. Several reasons may explain the difficulties in translation from animal stroke models to the human situation including open questions regarding the source of cells, the optimal dose of cells, the route of delivery and the time of treatment after the onset of ischemia. Considerable ethical constraints exist regarding the use of embryonic and fetal stem cells [14]. The preparation of autologous mesenchymal stem cells requires cell cultivation which might delay treatment initiation beyond a therapeutic time window within that therapy is effective [15,16]. Intravenous administration of cells is the least invasive method of delivery but transplanted cells only partly migrate to the infarcted brain [17]. A direct, intracerebral transplantation can reliable target a large number of cells closely to the infarct [18]. However, feasibility and safety issues remain, since surgical complications may occur. To meet the translational roadblocks we aimed to investigate the efficacy of a cell therapy in a rat stroke model that was shown to be logistically feasible and efficient in a clinical study of patients with myocardial infarction. The REPAIR-AMI trial is a large double-blind, randomized, multicenter study in which progenitor cells derived from bone marrow were intracoronary infused into the infarct artery 3 to 7 days after myocardial infarction [19]. The bone marrow mononuclear cells used in this study can be easily obtained by bone marrow aspiration without extensive preparation or cultivation before transplantation. As performed in the REPAIR-AMI trial we used an intraarterial injection for cell transplantation. This approach is routinely performed by neurointerventionalists for drug delivery and was shown to be feasibly and safe in acute stroke patients.

## Methods

### Photothrombotic ischemia model

All animal procedures were carried out in accordance with national and international regulations and approved by the local ethics committee. Experiments were performed on adult male Wistar rats (Charles River, Sulzfeld, Germany; 280 to 320 g body weight), which had free access to food and water. Animals were anesthetized with an intraperitoneal injection of ketamine hydrochloride (100 mg/kg body weight; Ketanest) and xylazine hydrochloride (8 mg/kg body weight; Ceva GmbH), and anesthesia was maintained if necessary. The left femoral vein was cannulated with PE-50 tube for Bengal Rose infusion. During the experiment, rectal temperature was maintained at 37°C by a thermostatically controlled heating pad (Föhr Medical Instruments). Photothrombotic ischemia was induced in the rat frontal cortex (right side) according to the method of Watson [20]. Animals were placed in a stereotaxic frame, and the scalp was incised for exposure of the skull surface. For illumination, a laser spot of 8 mm diameter (G Laser Technologies) was placed stereotaxically onto the skull 0.5 mm anterior to the bregma and 4 mm lateral from the midline. The skull was illuminated with a laser for 15.5 minutes. During the first minute of illumination, the dye rose bengal (0,133 mL/kg body weight, 10 mg/mL saline) was injected intravenously. After surgery, the catheter was removed and the animals were allowed to recover from the anesthesia.

### Donor cells

Bone marrow aspirates were obtained from two healthy volunteers in the cell-processing laboratory (Institute for Cardiovascular Regeneration, University of Frankfurt, Germany). The Ethics Review Board of the Hospital of the Johann Wolfgang Goethe University of Frankfurt, Germany, approved the protocol, and the study was conducted in accordance with the Declaration of Helsinki. Written informed consent was obtained from each volunteer. HBMC were isolated as described previously [21]. Briefly, bone marrow aspirates were diluted with 0.9% NaCl (1:5), were filtrated (100 μm) and mononuclear cells were isolated by density gradient centrifugation using Ficoll (Cambrex; 800 × g, 20 min, without brake). Mononuclear cells were washed for three times with 45 ml PBS (800 × g), were counted and sent by courier to the laboratory (Department of Neurology, University of Münster, Germany) where cell transplantations were performed.

### Intracarotid cell transplantation

Intracarotid injection of progenitor cells derived from human bone marrow (hBMC) was carried out 3 days after photothrombotic ischemia. Anesthesia was reinstituted and a midline neck incision was performed. The right common carotid artery (CCA), the external carotid artery (ECA) and the internal carotid artery (ICA) were exposed under an operating microscope (Novex Holland). The CCA and the ECA were ligated and a 5-0 suture was tied loosely at the origin of the ICA. A modified polyethylene catheter (PE-50, SIMS Portex Ltd) was inserted through an arteriectomy in the CCA and gently advanced into the ICA. Five million hBMC in 1 mL PBS (n = 9) or PBS alone (n = 7) were injected at 1 mL/min into each rat. Verum and control animals received daily injections of cyclosporine A (10 mg/kg, i.p.) throughout the experiment, commencing 24 hours before transplantation.

### Behavioral testing

Behavioral tests were performed before (baseline) and 2, 5, 14, 23 and 30 days after ischemia by an investigator (K.K.) who was blinded to the experimental groups. For Rotarod tests, rats were placed on an accelerating Rotarod cylinder, and the time the animals remained on the Rotarod was measured [22]. Speed was increased from 4 to 40 rpm within 5 minutes. The trial ended if the animal fell off the rungs or gripped the device and spun around for 2 consecutive revolutions without attempting to walk on the rungs. An arbitrary time limit of 600 seconds was set for rats on the Rotarod cylinder in training and testing procedures. The animals were trained 5 days before ischemia. The mean duration (seconds) on the device was recorded with 3 measurements 1 day before surgery. Motor test data are presented as percentage of mean duration (3 trials) on the Rotarod compared with the internal control (2 days after surgery).

For the adhesive-removal test, the somatosensory deficit was measured both before and after ischemia [23,24]. All rats were familiarized with the testing environment. In the initial test, 2 small pieces of adhesive-backed paper dots of equal size (113.1 mm^2^) were used as bilateral tactile stimuli occupying the distal-radial region at the wrist of each forelimb. The rat was then returned to a cage. The time to remove each stimulus from forelimbs was documented by 3 trials per day for each forepaw. Individual trials were separated by a time shift of at least 3 minutes. Before surgery, the animals were trained for 5 days. Once the rats were able to remove the dots within 10 seconds, they were subjected to ischemia. An asymmetry score was calculated as ratio of the difference of the impaired to the unimpaired forelimb to the sum of the impaired and the unimpaired forelimb: (time to remove ipsilateral dot - time to remove contralateral dot)/(time to remove ipsilateral dot + time to remove contralateral dot).

For the cylinder test, the rats were placed in a transparent cylinder (16 cm diameter, 21 cm high) and videotaped from below for 2 minutes [25]. Spontaneous wall and ground touches of the impaired contralateral forelimb and the hindpaw were counted. The rats were tested once before photothrombotic ischemia (baseline).

### Data and statistical analysis

Values are presented as mean ± SEM. Statistical analyses were carried out using the Statistical Package of Social Sciences (version 15.0, SPSS Inc., Chicago, IL). Sensorimotor measurements were analyzed with a 1-way ANOVA for each time point, followed by the post hoc Fisher protected least significance difference test. An α error rate of 0.05 was taken as the criterion for significance.

## Results

**Figure 1:**
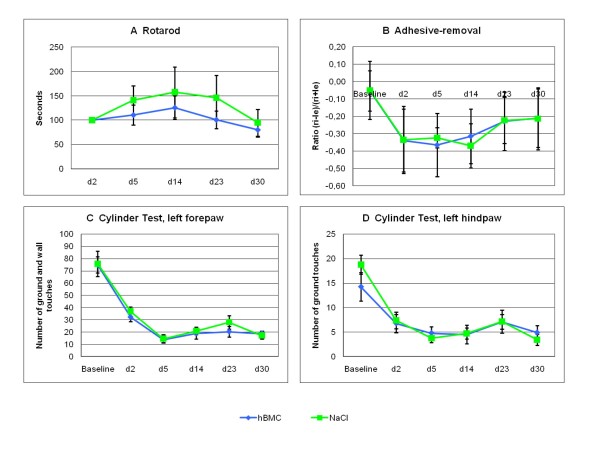
**Behavioral testing: Intraarterial treatment with hBMC (blue line) did not improve functional recovery after photothrombotic ischemia compared to placebo treatment (green line)**. Note the stable functional deficit over 30 days in both groups.

There was no significant difference in the temperature during surgery between cell-treated animals and controls. The percent body weight decline was 4% in the hBMC group and 8% in the placebo group (P > 0.05). Since the photothrombotic stroke model causes in somatosensory and motor deficits, we tested for both qualities. Motor recovery was assessed by the Rotarod test and the Cylinder test. Animals treated with hBMC had no favorable recovery regarding motor function (means ± SEM; P > 0.05, and means ± SEM; P > 0.05, ANOVA) as shown by figure 1. Somatosensory deficits also did not improve after hBMC treatment (means ± SEM; P > 0.05, ANOVA) compared to placebo treated rats (Figure 1).

## Discussion

The treatment paradigm in the present study was chosen on the basis of evidence from a clinical study that showed efficacy of hBMC's in patients with myocardial infarction. We showed that hBMC shipment from a cell-processing laboratory to a laboratory where cells are transplanted is feasible. We analyzed the long-term effects of an intraaterial hBMC treatment 3 days after photothrombotic stroke in rats using a battery of behavioral tests. Sensorimotor functions measured by the Rotarod test, the adhesive-removal test and the cylinder test were not improved after hBMC treatment compared to placebo. So far limited data exist regarding the efficacy of BMC's in animal stroke models. It was shown, that BMC's improved functional recovery when intraaterially injected 90 minutes (10 million cells per rat), 6 hours (20 million cells per rat) and 24 hours (4 or 10 million cells per rat) after the onset of ischemia [26-28]. A decreased efficacy after a delayed treatment initiation was reported after intravenous transplantation of BMC's [29,30]. This data suggest that hBMC treatment improves the outcome when transplanted early after stroke onset and that treatment three days after stroke as performed in our study is beyond the window of opportunity that allows functional recovery. The finding is of particular interest since systematic evaluations of the therapeutic time window after an intraarterial bone marrow stem cell treatment in animal studies are lacking so far. Nevertheless clinical trials were initiated that investigate bone marrow stem cell treatment in stroke patients at late time points between 9 and 90 days post-stroke (http://www.clinical trials.gov; NCT00761982, NCT00473057). The failed efficacy in our study emphasizes the importance of publishing negative preclinical studies to prevent the publication bias due to unpublished negative animal experimental studies which may contribute to an inadequate design of clinical trials. Moreover, the publication of negative results is necessary to perform meta-analyses which were shown to be an important tool to obtain a realistic impression of a treatment's efficacy in animal stroke studies [31,32].

Besides the late time point of transplantation another potential reason for the failed improved functional recovery after BMC treatment in our study might be the human origin of the cells. However, in a vascular disease model, the hindlimb ischemia model of the mouse, transplantation of human BMC's contributed to neovascularisation and improved outcome [33]. In contrast to our animal study BMC's in clinical studies would be of autologous origin and therefore no immunosuppression would be required.

## Conclusions

Our present study demonstrates that a treatment design with hBMC's that was shown to be successful in myocardial infarction cannot simply adopted for stroke therapy. A narrow window of opportunity to improve functional recovery after ischemic stroke might be the reason therefore.

## Competing interests

The authors declare that they have no competing interests.

## Authors' contributions

JM carried out the stroke induction, the cell transplantation procedures and wrote the manuscript. FHS carried out cell preparation and revised the manuscript. KK carried out functional recovery testing. KD carried out functional recovery testing and performed statistical analysis. MS revised the manuscript. SD carried out cell preparation. WRS participated in the design of the study and revised the manuscript.

## References

[B1] Lopez AD Mathers CD Ezzati M Jamison DT Murray CJ Global and regional burden of disease and risk factors, 2001: systematic analysis of population health data Lancet 2006 367 1747 1757 10.1016/S0140-6736(06)68770-9 16731270

[B2] Kolominsky-Rabas PL Heuschmann PU Marschall D Emmert M Baltzer N Neundorfer B Schoffski O Krobot KJ Lifetime cost of ischemic stroke in Germany: results and national projections from a population-based stroke registry: the Erlangen Stroke Project Stroke 2006 37 1179 1183 10.1161/01.STR.0000217450.21310.90 16574918

[B3] Dimmeler S Burchfield J Zeiher AM Cell-based therapy of myocardial infarction Arterioscler Thromb Vasc Biol 2008 28 208 216 10.1161/ATVBAHA.107.155317 17951319

[B4] Stem Cell Therapies as an Emerging Paradigm in Stroke (STEPS): bridging basic and clinical science for cellular and neurogenic factor therapy in treating stroke Stroke 2009 40 510 515 10.1161/STROKEAHA.108.526863 19095993

[B5] Bliss T Guzman R Daadi M Steinberg GK Cell transplantation therapy for stroke Stroke 2007 38 817 826 10.1161/01.STR.0000247888.25985.62 17261746

[B6] Chen J Sanberg PR Li Y Wang L Lu M Willing AE Sanchez-Ramos J Chopp M Intravenous administration of human umbilical cord blood reduces behavioral deficits after stroke in rats Stroke 2001 32 2682 2688 10.1161/hs1101.098367 11692034

[B7] Zhang J Li Y Zhang ZG Lu M Borneman J Buller B Savant-Bhonsale S Elias SB Chopp M Bone marrow stromal cells increase oligodendrogenesis after stroke J Cereb Blood Flow Metab 2009 10.1038/jcbfm.2009.41 PMC2849641 19384336

[B8] Giraldi-Guimaraes A Rezende-Lima M Bruno FP Mendez-Otero R Treatment with bone marrow mononuclear cells induces functional recovery and decreases neurodegeneration after sensorimotor cortical ischemia in rats Brain Res 2009 10.1016/j.brainres.2009.01.062 19368806

[B9] Jin K Mao X Xie L Galvan V Lai B Wang Y Gorostiza O Wang X Greenberg DA Transplantation of human neural precursor cells in Matrigel scaffolding improves outcome from focal cerebral ischemia after delayed postischemic treatment in rats J Cereb Blood Flow Metab 2009 10.1038/jcbfm.2009.219 PMC2831107 19826433

[B10] Caspi O Huber I Kehat I Habib M Arbel G Gepstein A Yankelson L Aronson D Beyar R Gepstein L Transplantation of human embryonic stem cell-derived cardiomyocytes improves myocardial performance in infarcted rat hearts J Am Coll Cardiol 2007 50 1884 1893 10.1016/j.jacc.2007.07.054 17980256

[B11] Mazo M Gavira JJ Abizanda G Moreno C Ecay M Soriano M Aranda P Collantes M Alegria E Merino J Transplantation of Mesenchymal Stem Cells exerts a greater long-term effect than Bone Marrow Mononuclear Cells in a chronic myocardial infarction model in rat Cell Transplant 2009 19919732 10.3727/096368909X480323

[B12] Borlongan CV Cell Therapy for Stroke. Remaining Issues to Address Before Embarking on Clinical Trials Stroke 2008 19064801 10.1161/STROKEAHA.108.533091 PMC4810678

[B13] Dimmeler S Burchfield J Zeiher AM Cell-based therapy of myocardial infarction Arterioscler Thromb Vasc Biol 2008 28 208 216 10.1161/ATVBAHA.107.155317 17951319

[B14] McLaren A Ethical and social considerations of stem cell research Nature 2001 414 129 131 10.1038/35102194 11689959

[B15] Chen J Sanberg PR Li Y Wang L Lu M Willing AE Sanchez-Ramos J Chopp M Intravenous administration of human umbilical cord blood reduces behavioral deficits after stroke in rats Stroke 2001 32 2682 2688 10.1161/hs1101.098367 11692034

[B16] Zhang J Li Y Zhang ZG Lu M Borneman J Buller B Savant-Bhonsale S Elias SB Chopp M Bone marrow stromal cells increase oligodendrogenesis after stroke J Cereb Blood Flow Metab 2009 10.1038/jcbfm.2009.41 PMC2849641 19384336

[B17] Hicks A Jolkkonen J Challenges and possibilities of intravascular cell therapy in stroke Acta Neurobiol Exp (Wars) 2009 69 1 11 19325636 10.55782/ane-2009-1724

[B18] Hayashi J Takagi Y Fukuda H Imazato T Nishimura M Fujimoto M Takahashi J Hashimoto N Nozaki K Primate embryonic stem cell-derived neuronal progenitors transplanted into ischemic brain J Cereb Blood Flow Metab 2006 26 906 914 10.1038/sj.jcbfm.9600247 16395293

[B19] Schachinger V Erbs S Elsasser A Haberbosch W Hambrecht R Holschermann H Yu J Corti R Mathey DG Hamm CW Intracoronary bone marrow-derived progenitor cells in acute myocardial infarction N Engl J Med 2006 355 1210 1221 10.1056/NEJMoa060186 16990384

[B20] Watson BD Dietrich WD Busto R Wachtel MS Ginsberg MD Induction of reproducible brain infarction by photochemically initiated thrombosis Ann Neurol 1985 17 497 504 10.1002/ana.410170513 4004172

[B21] Schachinger V Erbs S Elsasser A Haberbosch W Hambrecht R Holschermann H Yu J Corti R Mathey DG Hamm CW Intracoronary bone marrow-derived progenitor cells in acute myocardial infarction N Engl J Med 2006 355 1210 1221 10.1056/NEJMoa060186 16990384

[B22] Hamm RJ Pike BR O'Dell DM Lyeth BG Jenkins LW The rotarod test: an evaluation of its effectiveness in assessing motor deficits following traumatic brain injury J Neurotrauma 1994 11 187 196 10.1089/neu.1994.11.187 7932797

[B23] Schallert T Kozlowski DA Humm JL Cocke RR Use-dependent structural events in recovery of function Adv Neurol 1997 73 229 238 8959217

[B24] Schabitz WR Berger C Kollmar R Seitz M Tanay E Kiessling M Schwab S Sommer C Effect of brain-derived neurotrophic factor treatment and forced arm use on functional motor recovery after small cortical ischemia Stroke 2004 35 992 997 10.1161/01.STR.0000119754.85848.0D 14988579

[B25] Hicks AU MacLellan CL Chernenko GA Corbett D Long-term assessment of enriched housing and subventricular zone derived cell transplantation after focal ischemia in rats Brain Res 2008 1231 103 112 10.1016/j.brainres.2008.07.041 18675262

[B26] Kamiya N Ueda M Igarashi H Nishiyama Y Suda S Inaba T Katayama Y Intra-arterial transplantation of bone marrow mononuclear cells immediately after reperfusion decreases brain injury after focal ischemia in rats Life Sci 2008 83 433 437 10.1016/j.lfs.2008.07.018 18727931

[B27] Baker AH Sica V Work LM Williams-Ignarro S de Nigris F Lerman LO Casamassimi A Lanza A Schiano C Rienzo M Brain protection using autologous bone marrow cell, metalloproteinase inhibitors, and metabolic treatment in cerebral ischemia Proc Natl Acad Sci USA 2007 104 3597 3602 10.1073/pnas.0611112104 17360688 PMC1805552

[B28] Brenneman M Sharma S Harting M Strong R Cox CS Jr Aronowski J Grotta JC Savitz SI Autologous bone marrow mononuclear cells enhance recovery after acute ischemic stroke in young and middle-aged rats J Cereb Blood Flow Metab 2009 19773802 10.1038/jcbfm.2009.198 PMC2893568

[B29] Iihoshi S Honmou O Houkin K Hashi K Kocsis JD A therapeutic window for intravenous administration of autologous bone marrow after cerebral ischemia in adult rats Brain Res 2004 1007 1 9 10.1016/j.brainres.2003.09.084 15064130

[B30] Giraldi-Guimaraes A Rezende-Lima M Bruno FP Mendez-Otero R Treatment with bone marrow mononuclear cells induces functional recovery and decreases neurodegeneration after sensorimotor cortical ischemia in rats Brain Res 2009 10.1016/j.brainres.2009.01.062 19368806

[B31] Minnerup J Schabitz WR Multifunctional actions of approved and candidate stroke drugs Neurotherapeutics 2009 6 43 52 10.1016/j.nurt.2008.10.032 19110198 PMC5084255

[B32] Minnerup J Heidrich J Wellmann J Rogalewski A Schneider A Schabitz WR Meta-analysis of the efficacy of granulocyte-colony stimulating factor in animal models of focal cerebral ischemia Stroke 2008 39 1855 1861 10.1161/STROKEAHA.107.506816 18403735

[B33] Heeschen C Lehmann R Honold J Assmus B Aicher A Walter DH Martin H Zeiher AM Dimmeler S Profoundly reduced neovascularization capacity of bone marrow mononuclear cells derived from patients with chronic ischemic heart disease Circulation 2004 109 1615 1622 10.1161/01.CIR.0000124476.32871.E3 15037527

